# Effectiveness of a hydrophobic dressing for microorganisms’ colonization of vascular ulcers: Protocol for a randomized controlled trial (CUCO‐UV Study)

**DOI:** 10.1111/jan.14412

**Published:** 2020-06-08

**Authors:** Juan C. Morilla‐Herrera, José M. Morales‐Asencio, Alberto J. Gómez‐González, Antonio Díez‐De Los Ríos, Inmaculada Lupiáñez‐Pérez, Carlos Acosta‐Andrade, Marta Aranda‐Gallardo, Ana B. Moya‐Suárez, Shakira Kaknani‐Uttumchandani, Silvia García‐Mayor

**Affiliations:** ^1^ Department of Nursing University of Málaga, Faculty of Health Sciences Malaga Spain; ^2^ Distrito Sanitario Málaga – Valle del Guadalhorce Malaga Spain; ^3^ Instituto de Investigación Biomédica de Málaga (IBIMA) Malaga Spain; ^4^ Agencia Sanitaria Costa del Sol Marbella Spain

**Keywords:** bacterial adhesion, bacterial load, leg injuries, nursing, varicose ulcer, wound infections

## Abstract

**Aim:**

To determine the effectiveness of a hydrophobic dressing (Cutimed Sorbact^®^) against a silver dressing (Aquacel^®^ Ag Extra) in the level of colonization of chronic venous leg ulcers. The secondary endpoints are health‐related quality of life, level of pain, and time to complete healing.

**Design:**

Open randomized controlled trial, with blinded endpoint.

**Methods:**

Patients with chronic venous leg ulcers with signs of critical colonization will be randomized in a concealed sequence using computer software to receive one of the alternative dressings. A total of 204 participants recruited in Primary Health Care and nursing homes will be necessary to assure statistical power. Measures will include sociodemographic variables, wound‐related variables (area, exudate, and time to healing), level of pain, adverse effects, and health‐related quality of life. Smear samples will be collected from the ulcers and will be subject to DNA‐typing technique through polymerase chain reaction to obtain the level of colony‐forming units. Measures will be collected at baseline, 4, 8, and 12 weeks.

**Discussion:**

Elevated levels of microorganisms prevent wound healing and favour its chronification. The main target when colonization is present is to reduce the bacterial load to levels that promote immune system mobilization. Hydrophobic dressings prevent the formation of biofilm in the wound by means of physical effect, so that the possibility of antimicrobial resistance is significantly reduced.

**Impact:**

Current evidence about the effectiveness of dressings to minimize venous leg ulcers colonization is very limited. Previous studies have important methodological flaws. This study will permit to obtain the effectiveness of hydrophobic dressings against silver dressings with a robust design based on conditions of routine clinical practice in Primary Health Care and nursing homes.

## Abstract (zh)

**目的:**

本研究旨在测定疏水性敷料(Cutimed Sorbact®)与含银敷料(Aquacel®Ag Extra)在下肢慢性静脉性溃疡的细菌定植水平方面的有效性。次要终点包括:健康相关生活质量、疼痛程度和完全愈合时间。

**设计:**

开放式随机对照试验(盲终点)。

**方法:**

利用计算机软件,按照隐性顺序,安排有严重细菌定植迹象的下肢慢性静脉性溃疡患者随机接受其中一种替代敷料。为保证统计功效,需要在初级保健护理院和养老院共招募204名受试者。衡量指标将包括社会人口统计变量、伤口相关变量(伤口大小、渗出物和愈合时间)、疼痛程度、不良反应和与健康相关生活质量。从溃疡处采集涂片样本,通过聚合酶链反应,采用DNA分型技术,对涂片样本进行分析,以获得菌落形成单位的水平。在基准日期、4周、8周和12周收集测量值。

**讨论:**

微生物水平升高会阻碍伤口愈合,会促成疼痛迁延化。当出现定植时,主要目标是将菌载量降低至促进免疫系统动员的水平。疏水性敷料通过物理作用防止伤口内形成生物膜,从而明显降低耐药的可能性。

**影响:**

目前有关敷料最大限度地减少下肢静脉溃疡定植的有效性的证据非常有限。以往的研究在方法上存在重大缺陷。本研究将允许根据初级保健护理院和养老院的常规临床实践条件,通过可靠设计,获得疏水性敷料和含银敷料的有效性。

## 1 INTRODUCTION

Chronic wounds of torpid evolution are currently a great challenge for health services worldwide. In Europe, they can eventually lead to 2–4% of health expenditure (Posnett, Gottrup, Lundgren, & Saal, 2009). Particularly, venous ulcers are estimated that would affect at least 1% of adult population throughout their lifetime. Studies have determined venous ulcer frequency around 0.2–1.1% (Graham, Harrison, Nelson, Lorimer, & Fisher, 2003), thought this figure can rise up to 4% in people over 65 years old (Pugliese, 2016).

Clinical nursing has a key role in the detection, diagnosis, treatment, and tracking of venous leg ulcers. It is important to identify the different types of wounds and their characteristics that nurses can find in their clinical practice, and the appropriate treatments that can be carried on. Furthermore, the healing of these chronic wounds depends on the choice of the most suitable designed dressing. Most of times, this decision relies on the clinical nurse's judgment about the venous ulcer evolution (Davies, Turton, Woolfrey, Elley, & Taylor, 2005).

### 1.1 Background

More than 80% of venous leg ulcers may be colonized or infected by bacteria (O’Meara et al., 2014). One of the leading causes of delay in the healing is the presence of pathogenic bioburden. In fact, bacterial biofilms (exopolysaccharides extracellular matrix which provides structural bacterial defence) over the wound have been associated with chronic wound interference recovery (Clinton & Carter, 2015; Gompelman, van Asten, & Peters, 2016; Hurlow et al., 2015). Moreover, biofilm provides a key resistance to local and systemic antibiotic treatments, especially in the case of venous ulcers (Bianchi et al., 2016). Major representative strains are *Staphylococcus aureus* and *Pseudomonas aeruginosa*, while it can be also found in anaerobic microorganisms (*Peptostreptococcus* species and gram‐negative *Bacillus*), *Enterobacteria,* or group A *Streptococcus* (Etchebarne et al., 2017).

The presence of colonization or infection in chronic wounds produces a direct effect in their healing and recovery when colony‐forming units (CFUs) exceed 10^5^ per tissue gram. Thus, wound colonization by pathogenic bacteria promotes its chronification. Furthermore, pathogenic bacteria can also generates intra‐wound synergistic effects with other non‐pathogenic microorganisms increasing their action (Rahim et al., 2017).

This problem has been usually addressed by using local dressings, with the aim to reduce the total wound bacterial load. Although the most common therapeutic strategy is the use of silver dressings due to their antimicrobial effect, there is a lack of evidence about their effectiveness in venous ulcers (O’Meara et al., 2014). Moreover, there is some uncertainty over adverse effects derived from systemic absorption of silver molecules, in addition to generate silver resistance in many bacteria strains (Maillard & Hartemann, 2013; Mijnendonckx, Leys, Mahillon, Silver, & Van Houdt, 2013; Percival et al., 2008). Additionally, these inconveniences can involve an elevated cost when long‐term treatments are needed.

A novel approach to deal with the problem of chronic wound colonization is the use of hydrophobic dressings. It has been extensively addressed that when two hydrophobic molecules contact each other, they increase their molecular entropy by hydrophobic interactions to finally expelling residual water molecules (Curtis, Steinbrecher, Heinemann, Blanch, & Prausnitz, 2002). Thus, hydrophobic surface microorganisms present a high probability to join to hydrophobic dressings, allowing hydrophilic bacteria to rest at the wound bed to promote the healing. In vitro experiments have also showed that most pathogenic microorganisms present high hydrophobic membrane molecules (Ljungh, Yanagisawa, & Wadström, 2006).

Hydrophobic mechanism is neither based on active absorption of exudate nor placing chemical agents on the surface of the ulcer. In fact, the presence of dialkylcarbamoyl chloride (DACC)‐coated dressings attract hydrophobic fungus and bacteria, avoiding the formation of biofilm in the wound (Derbyshire, 2010). In this sense, antimicrobial properties of hydrophobic dressings are based on physical effects instead of chemical effects. Consequently, the possibility to generate antimicrobial resistance is significantly reduced (Ljungh et al., 2006). Furthermore, it has been analysed how local antiseptic administration could affect to the expression of the hydrophobic bacterial membrane. Thus, the use of eutectic mixture of local anaesthetics (EMLA) or glycol‐carbohydrates modified polymers (Askina^®^) can interfere hydrophobicity, whereas this property is not affected by other products commonly used in the care of wounds, such as povidone‐iodine or saline solutions (Ljungh et al., 2006).

Although there are some preliminary studies which compare high hydrophobic dressings versus silver dressings, they have been carried out with reduced samples in pilot studies (Mosti, Magliaro, Mattaliano, Picerni, & Angelotti, 2015; Mussi & Salvioli, 2004). Furthermore, the study population in these studies did not include patients in home or ambulatory care, the most frequent clinical settings with a high demand for this type of wounds. In addition, the trials carried out have evaluated the results throughout short follow‐up periods. All these issues limit the available evidence on hydrophobic dressings, as it has been stated in a recent systematic review (Totty et al., 2017).

## 2 THE STUDY

### 2.1 Aim

The main aim of the study is to determine the effectiveness of a hydrophobic dressing (Cutimed Sorbact^®^) against a silver dressing (Aquacel^®^ Ag Extra) in the level of colonization of chronic venous leg ulcers.

The secondary aims will be to analyse the effect of Cutimed Sorbact^®^ versus Aquacel^®^ Ag Extra on total healing, the proportion of wound reduction, time of recovery, level of pain during recovery, and health‐related quality of life. In addition, the safety of Cutimed Sorbact^®^ will be evaluated.

The null hypothesis is that there is no difference in the level of bacterial colonization at 12 weeks of follow‐up in chronic ulcers treated with Cutimed Sorbact^®^ versus those treated with Aquacel^®^ Ag Extra.

### 2.2 Methods

#### 2.2.1 Design

An open, randomized, controlled trial with blinded evaluators will be carried out.

#### 2.2.2 Sample

Assuming a 95% confidence level and 80% statistical power, 186 participants would be necessary (93 for each study arm) to detect a 20% load bacteria reduction (Mosti et al., 2015). This sample will be increased up to 204 participants to cover any possible patient dropouts.

#### 2.2.3 Allocation

Inclusion of participants will be randomized in a concealed sequence to participants, clinical nurses, and investigators. Once a participant meets the inclusion criteria, collaborative investigators will know by means of a telephone call the study group assigned according to the random sequence. The random sequence will be generated by a computerized system. Although the study groups are not blinded for patients and nurses, the members of the team who will carry out the analyses will be blinded to the allocation groups.

#### 2.2.4 Participants

##### Inclusion criteria

The study will be developed in adult patients who are attended in health centres or nursing homes belonging to the District of Primary Health Care of Málaga (Spain), who present a chronic venous vascular ulcer located in their lower limbs with signs of critical colonization, according to the criteria of Lazareth and Moore (Lazareth et al., 2007; Moore, 2013). These criteria imply the presence of at least three of the following: (a) Severe pain during dressing change; (b) Perilesional oedema; (c) Local oedema; (d) Unpleasant smell; and (e) Abundant pus.

In case of fulfilling the mentioned criteria, an exudate sample will be collected from the wound by the primary healthcare nurse. Ulcer infection would be assumed in case of a level of microorganisms per tissue gram or microbial counting over 10^5^ CFUs per microlitre (μL) (Pugliese, 2016).

##### Exclusion criteria

The exclusion criteria will be: people younger than 18 years old, venous ulcer that does not meet Lazareth and Moore criteria, arterial ulcers, participants with diabetes and without pedis pulse, people who are immunosuppressed, patient with rheumatoid arthritis in the acute phase, patients with dermatitis prior to the appearance of the ulcer, and patients with neuropathy or lack of sensitivity of any aetiology.

#### 2.2.5 Data collection

Patients will be recruited by clinical nurses in health centres and nursing homes belonging to the District of Primary Health Care of Málaga (Spain). Following obtaining the patients’ informed consent, sociodemographic variables (age, gender), wound location and characteristics, and current treatment will be collected at baseline, together with the endpoints by the nurse who usually give care to the patient. This will reinforce the patient adherence to the study.

##### Primary endpoint

Level of colonization: it will be evaluated by quantitative real‐time polymerase chain reaction (qPCR) identification technique and quantified in CFUs and in nanograms (ng) of bacterial deoxyribonucleic acid (DNA) per μl of vascular ulcer exudate.

Two samples per wound (to have a reserve sample in case of possible contamination) will be collected by cotton swab directly from the wound bed. The samples will be collected before cleaning the wound to obtain a reliable microorganisms’ concentration. Swab tip will be rotated in a 1 cm^2^ wound surrounding area for 5 s. Next, cotton swabs will be placed in a sterile container at 4 ºC (Pugliese, 2016) until the process of qPCR.

Then, to determinate the total microorganisms’ quantity, samples will be subjected to a thermal shock, which consists in freezing them at −80ºC for 5 min, followed by heating samples at 95ºC for additional 5 min. With this procedure, gram‐positive and gram‐negative bacteria will be efficiently lysed. After spin centrifugation, DNA strands will be extracted for the following steps. The total DNA quantity of the isolated DNA will be measured using NanoDrop^TM^ spectrophotometer. Microorganisms will be quantitative determined by qPCR (referred to number of DNA copies amplified by this technique) (Rhoads, Wolcott, Sun, & Dowd, 2012).

Fast EvaGreen^®^ qPCR Master Mix (2X) kit will be used for qPCR amplification reactions, including specific primers usually present in vascular ulcers (Table 1; Etchebarne et al., 2017). Serial dilution will be performed from purified genomic DNA to generate a concentration calibration curve to compare data obtained from samples. From there, it will be possible to detect not only the presence of microbial species but also the quantitative determination of variation in these stains in samples of the same patient, as well as comparing treatments. Results will be standardized to CFUs per μL of exudate.

**Table 1:** Pathogen list usually founded and detected in vascular ulcers (Majewski et al., 1995) in addition to multi‐resistant pathogens (Moore et al., 2010)

Microorganism	Feature
*Staphylococcus aureus*	Commonly found on vascular ulcers
*Corynebacterium striatum*	
*Pseudomonas aeruginosa*	
*Helcococcus kunzii*	
*Proteus mirabilis*	
*Finegoldia magna*	
*Escherichia coli*	
*Staphylococcus aureus* methicillin/oxacillin resistant	Multi‐resistant pathogen described on vascular ulcers
*Acinetobacter baumannii* MDR (Imipenem resistant)	
*Klebsiella pneumoniae* producing BLEE	
*Enterococcus* vancomycin resistant	
*Enterobacteria* producing carbapenem	
*Pseudomonas aeruginosa* MDR producing BLEE or carbapenem, or piperacillin resistant	

##### Secondary outcome measures

Wound size will be evaluated by planimetry using PictZar^®^ 7.6.1 software (Wendelken et al., 2011). Photos will be taken from affected area completely in parallel with respect to the ulcer and the photo will have to include a calibrated rule of 3 cm (standardized procedure according to PictZar^®^ planimetry). The characteristics and clinical evolution of the wound will be evaluated using the Resvech 2.0 scale (Restrepo‐Medrano & Verdú Soriano, 2011).

Healing time will be measured by the number of days until complete healing. This outcome will be complied if re‐epithelialization of the skin is confirmed in two consecutive visits across 2 weeks. Pain will be measured using the Numerical Rating Scale (NRS).

Health‐related quality of life will be measured by means of the Charing Cross Venous Ulcer Questionnaire (CCVUQ) (González de la Torre, Quintana‐Lorenzo, Perdomo‐Pérez, & Verdú, 2017; González‐Consuegra & Verdú, 2010). Adverse events related to the treatment will be collected both by nurses and patients. Follow‐up will be carried out at 4, 8, and 12 weeks, except the quality of life, which will be only evaluated at baseline and 12 weeks. Adherence to the treatment will be checked with the nurse and with the evaluation of consumed dressings.

#### 2.2.6 Intervention

Following the obtention of the baseline data, the nurse who usually gives care to the patient will proceed to cure the venous ulcer using a standardized procedure. They will wash the area with saline solution and evaluate if mechanical debridement is necessary. Following, they will apply Cutimed Sorbact^®^ dressing (intervention) or Aquacel^®^ Ag Extra (control) dressings in the wound. Finally, they will cover the wound with a secondary gauze dressing and a double compression bandage. Patient's appointments will not extend beyond 72 hr, although depending on the level of exudate and the status of the wound, the nurse may decide to shorten this interval.

Dropout criteria are as follows: (a) Local or systemic clinical deterioration that indicates that continuation at the study is not appropriate; (b) Lack of patient's concordance; (c) Withdrawal of patient consent; and (d) Complete healing before week 12. The data collection period comprehends from May 2012 to December 2020.

#### 2.2.7 Data analysis

Data analysis will include descriptive statistics and exploratory analysis to determine the normality of the distributions and to check the range of data values. Bivariant analysis will be performed by chi‐square test, Student *t* test, Mann–Whitney U, Wilcoxon W, ANOVA, and Kruskal–Wallis test, depending on the normality of the distributions, type of variable analysed, and their homoscedasticity, which will be tested by the Levene's test. Intra‐group analysis will be performed by general linear models, with the corresponding corrections in multiple comparisons by Bonferroni test. To test the null hypothesis (inter‐group analysis), lineal generalized models will be developed in repeated measures, taking on consideration CFUs number as dependent variable as well as control or experimental designation group as a factor. Even if basal differences are significative, they will be attached as adjustment factor, following Mallinckrodt and Lipkovich recommendations (Mallinckrodt, Lipkovich, & Lipkovich, 2016). Sphericity will be tested using Mauchly test, Greenhouse‐Geisser test, or Huynh‐Feldt test in case of no setting.

All analyses will be performed by an intention‐to‐treat approach. To this purpose, missing data will be calculated by multiple imputation methods.

All the analyses will be performed in a blinded way.

#### 2.2.8 Ethical considerations

The study has been approved by the Ethics and Research Committee of Malaga. The study will meet the requirements of Helsinki Statement and later revisions and the principles of good clinical practices. Informed consent will be demanded to the participants in accordance with current law in Spain by using an application form and an information sheet designed for this purpose. Due to the characteristics of the target study population, it is possible to find patients with presence of cognitive impairment. In those cases, informed consent will be requested from their caregivers.

The Ethics and Research Committee of Malaga will be reported in advance on any modification in the eligibility criteria, outcomes, or analyses. All the monitoring procedure of adverse effects will be driven by the regulation referred to MEDDEV 2.7/3 Guide Review 3 “Clinical investigations: Serious adverse event reporting under Directives 90/385/EEC and 93/42/EEC” and by the ISO regulation number 14155:2012. All the participants potential harms will be covered by an insurance policy issued for the trial. This trial is registered at Clinical Trials (NCT03667937).

#### 2.2.9 Validity and reliability

According to ISO 14971 regulation, several procedures have been set to manage potential risk due to the use of the study products. Scientific literature has been extensively reviewed about risks of Cutimed Sorbact^®^ dressings, with no adverse effects reported to date (Totty et al., 2017).

A key strength of this study is that it will be performed under routine clinical conditions, in multiple centres, both in Primary Health Care and nursing homes. This feature will give a strong external validity. In addition, the use of planimetry software PictZar^®^ ensures objectivity to evaluate the wound area.

The trial will be supervised by an independent monitoring committee integrated by external members belonging to the District of Primary Health Care of Málaga and IBIMA. This committee will carry out audits each 6 months. On the other hand, some potential confounding variables will be recorded such as antibiotic and/or analgesic therapy, nutritional status, and level of dependence (Barthel index). Moreover, this trial will comply with a rigorous procedure to report adverse events, to avoid the usual lack of reporting adverse events in venous leg ulcer research (Jull & Biggs, 2019).

## 3 DISCUSSION

The prevalence of venous leg ulcers tends to raise due to its association with ageing. In Western countries chronic venous conditions consume up to 2% of healthcare budget (Davies, 2019). The impact of venous leg ulcers on quality of life affects multiple dimensions such as difficulties with activities of daily living, social isolation, pain, or disability (Joaquim, Silva, Garcia‐Caro, Cruz‐Quintana, & Pereira, 2018). Multiple approaches are available using local dressings to treat venous leg ulcers combined with other interventions (O’Meara et al., 2014). Bacterial colonization or infection is one of the complications that hinder an adequate healing, since wound bed microorganisms increase their resistance by using a biofilm (Maillard & Hartemann, 2013; Mijnendonckx et al., 2013).

This trial attempts to evaluate a physical‐based approach instead of the current chemical one based on silver dressings. The use dressings that mimic biofilm conditions, try to produce a bacterial migration into the dressing. Thus, no chemical intervention in the wound bed is carried out, avoiding possible side effects (Ljungh et al., 2006). It is important to note that either high levels or no presence of microorganisms do not help to wound healing and favour its chronification. Consequently, the main aim of these dressings is not to eliminate the bacterial load, but to reduce it to limited levels to activate the immune system at the wound site, providing growth factors that facilitate wound healing (Ojalvo et al., 2017).

If the results of this trial show higher effectiveness of Cutimed Sorbact^®^, in terms of reduced colonization, without adverse events, it is presumable that this turns into shorter healing times, with a substantial reduction in the use of health staff time. Additionally, the study will be able to evaluate the impact on health‐related quality in aspects such as pain, exudate, odour, mobility, and activities of daily living, or personal relationships (Phillips et al., 2018).

The final expected results of this study could have many advantages on nurse's clinical practice. Due to the approach of avoiding chemical wound treatment, the use of broad‐range antibiotics may be reduced significantly. As a consequence, this approach could lead to a decrease in the emergence of most common multi‐resistant microorganisms in hospital services. Besides, physical‐based dressing approach would imply an important change on nurse research in wounds, leaving out the use of chemical molecules in intervention strategies (Moeini, Pedram, Makvandi, Malinconico, & Gomez d’Ayala, 2020).

### 3.1 Limitations

This study does not include patients with venous ulcers and compromised distal arterial blood circulation, or lack of sensitivity of any aetiology in their lower limbs. Further studies should be carried out in people with these clinical features to evaluate the effectiveness of Cutimed Sorbact^®^ against Aquacel^®^ Ag Extra. Likewise, those patients with rheumatoid arthritis in an acute exacerbation phase, or those with local dermatitis prior to the existence of the ulcer will not be included.

## 4 CONCLUSIONS

New approaches are required to improve venous ulcers treatment to avoid antimicrobial resistance and limit the use of chemical agents. One of these new approaches would be the use of hydrophobic dressings. A large sample and routine conditions of clinical practice will give this study a strong external validity and generalizability.

## CONFLICT OF INTEREST

None of the authors or participating researchers in the study have potential conflict of interest regarding consultancy, employment, advocacy groups, grants, fees and honoraria, patents, royalties, and stock or share ownership with BSN Medical. The agreement between IBIMA and BSN Medical included a clause of “No Inducement” that represents the fair market value for the services provided and that Funder's support provided under this agreement imposes no obligation, express or implied, for IBIMA or principal investigators to purchase, prescribe, refer, provide favourable status for, or otherwise support funder's products and services.
